# The Relationship Between Childhood Trauma and the Response to Group Cognitive-Behavioural Therapy for Chronic Fatigue Syndrome

**DOI:** 10.3389/fpsyt.2020.00536

**Published:** 2020-06-12

**Authors:** Maud De Venter, Jela Illegems, Rita Van Royen, Bernard G. C. Sabbe, Greta Moorkens, Filip Van Den Eede

**Affiliations:** ^1^University Psychiatric Department, Campus Antwerp University Hospital (UZA), Edegem, Belgium; ^2^Faculty of Medicine and Health Sciences, Collaborative Antwerp Psychiatric Research Institute (CAPRI), University of Antwerp (UA), Antwerp, Belgium; ^3^Behaviour Therapy Division for Fatigue and Functional Symptoms, Antwerp University Hospital (UZA), Edegem, Belgium; ^4^Department of Internal Medicine, Antwerp University Hospital (UZA), Edegem, Belgium

**Keywords:** chronic fatigue syndrome, childhood trauma, group cognitive-behavioural therapy, treatment outcome, physical functioning, myalgic encephalomyelitis

## Abstract

**Objective:**

To examine the relationship between childhood trauma and the response to group cognitive-behavioural therapy (GCBT) for chronic fatigue syndrome (CFS).

**Methods:**

A single cohort study conducted in an outpatient university referral center for CFS including a well-documented sample of adult patients meeting the CDC criteria for CFS and having received 9 to 12 months of GCBT. A mixed effect model was adopted to examine the impact of childhood trauma on the treatment response in general and over time. The main outcome measures were changes in fatigue, as assessed with the Checklist Individual Strength (total score), and physical functioning, as gauged with the Short Form 36 Health Survey subscale, with the scales being completed at baseline, immediately after treatment completion and after 1 year.

**Results:**

We included 105 patients with CFS. Childhood trauma was not significantly associated with the response to GCBT over time on level of fatigue or physical functioning.

**Conclusion:**

Childhood trauma does not seem to have an effect on the treatment response to dedicated GCBT for CFS sufferers over time. Therefore, in the allocation of patients to this kind of treatment, a history of childhood trauma should not be seen as prohibitive.

## Introduction

According to Fukuda et al. chronic fatigue syndrome (CFS) is characterised by “clinically evaluated, unexplained, persistent or relapsing chronic fatigue that is of new or definite onset (has not been lifelong); is not the result of ongoing exertion; is not substantially alleviated by rest; and results in substantial reduction in previous levels of occupational, educational, social, or personal activities.” For a formal diagnosis of CFS, at least four of the following symptoms should also have been present concurrently during at least 6 successive months: sore throat, muscle pain (myalgia), multi-joint pain, tender cervical or axillary lymph nodes, post-exertion malaise, unrefreshing sleep, new headaches, impaired memory or concentration (1). Prevalence estimates of CFS range from 0.2% to 6.4% and is more common in women than in men (2). In 2015, the Committee on the Diagnostic Criteria for Myalgic Encephalomyelitis/CFS of the Institute of Medicine proposed to replace the name Myalgic Encephalomyelitis/CFS by Systemic Exertion Intolerance Disease (SEID). New diagnostic criteria were introduced, requiring: “A substantial reduction or impairment in the ability to engage in preillness levels of occupational, educational, social, or personal activities that persists for more than 6 months and is accompanied by fatigue, which is often profound, is of new or definite onset (not lifelong), is not the result of ongoing excessive exertion, and is not substantially alleviated by rest”, as well as the major criteria postexertional malaise and unrefreshing sleep and either cognitive impairment or orthostatic intolerance (3, 4).

For a long time, the guideline-recommended treatments for CFS across countries have been graded exercise therapy (GET) and cognitive-behavioural therapy (CBT). In CBT for CFS the focus is on thought patterns about chronic fatigue, the impact on the symptoms and management of those symptoms. The goal is to gradually increase the level of activity of the patient. Findings from a large RCT evaluating group CBT (GCBT) for CFS suggest that patients with CFS can be treated effectively in groups, independently of group size (5). Importantly, a recent study on the long-term effects (> 5 years) of different forms of CBT for CFS (individual, stepped-care, and group formats) found enduring partial improvement of fatigue severity and physical functioning regardless of the type of treatment, although the small number of patients being treated and thus the limited power of the study need mentioning (6).

CFS clearly is a very heterogeneous condition both with respect to clinical presentations and underlying disease pathways (7, 8). There hence is an urgent need for research to identify and investigate mediators and moderators of the clinical course and treatment outcomes of somatic symptom disorders, bodily distress disorders and functional disorders (9). Research into differential predictors of the effects of CBT will optimise its indication statement, implementation, and response to personalised treatment. Evidently, a critical attitude towards CFS treatments whose effects were previously taken for granted is called for.

Previous studies emphasise that childhood trauma is an important predisposing factor in CFS (8) and is associated with an approximate fourfold risk of developing the disorder, even when accounting for comorbid psychiatric disorders (10–14). In a recent study of our research group on the impact of childhood trauma subtypes, sexual harassment emerged as the most important influencing factor of fatigue and poor physical functioning (15). Unfortunately, there is limited research on the persistent influence of childhood trauma during and after CBT, although this may well be of high clinical importance. More precisely, childhood trauma has previously been associated with reduced functioning of the hypothalamic-pituitary-adrenal (HPA) axis in CFS (13, 16) and, in turn, hypocorticolism has been shown to predict a poor response to CBT (17).

However, evaluating individual CBT, Heins et al. (18) found no differences in fatigue changes between patients with or without a history of childhood maltreatment during the intervention or at their 6-month follow-up. Notably, given their dichotomous approach, their use of strict cut-off scores of childhood maltreatment, and the omission to gauge the emotional experience of the adverse event(s) reported, these results should be interpreted with caution. To add to our knowledge, in the current study we examine the short- and longer-term effects (up to 12 months) of childhood trauma on fatigue and physical-functioning outcomes of GCBT in a well-defined cohort of CFS patients while taking the duration of the trauma, the victim’s relationship with the perpetrator, and the subjective response to the trauma into account.

## Material and Methods

### Design and Participants

We conducted a single cohort study to assess the impact of childhood trauma on the response of adult CFS sufferers to a dedicated GCBT programme. From 2004 until 2008, 1,596 patients have been referred to our centre by their general practitioners on suspicion of CFS of which 571 (35.8%) turned out to be eligible for GCBT and 475 (29.8%) agreed for participation in the treatment programme. The timeframe between the therapeutic intake procedure and the 12 months follow-up was on average 2 to 3 years. Within the year after the intake procedure participants started the group treatment programme. The data collection was conducted before and at the end of treatment, and after 1 year.

All participants of the group programme were seen by an experienced internist (GM), who confirmed the diagnosis according to the 1994 case definition (panel) (1) as based on serial physical examinations and laboratory measurements. To be eligible for therapy, patients had to be Dutch-speaking and between 18 and 65 years old. Eligible patients were subsequently interviewed by a psychiatrist (FVDE) or a (supervised) psychiatry trainee in accordance with the Dutch version of the Structured Clinical Interview for DSM-IV Axis I Disorders, version 2.0 (19). The psychiatric exclusion criteria for CFS as formulated by the 1994 case definition were adopted (1). Patients were then referred for a therapeutic intake procedure. Before inclusion in the treatment programme, the following criteria were also comprehensively evaluated and discussed with the patient: physical capacities, distance to the treatment centre, mobility, family situation, work, the possibility to practise new behaviours, and major life events expected in the next year. Participation in the group intervention was voluntary and fully covered by federal health insurance (RIZIV/INAMI, 2002).

In a first study phase, adult CFS patients who completed the treatment programme were invited by letter to take part in genetic and trauma-related research in CFS (20). For the recruitment of this study, 168 patients originally responded to an invitation to participate and attended the test sessions (we organized 12 sessions between July 2007–May 2010). Eight patients did not fully complete the Traumatic Experiences Checklist (TEC) and were excluded, leaving the data of 160 patients. The study protocol was approved by the Medical Ethics Committee of the Antwerp University Hospital (file code: 7/1/4; date: 27/2/2007).

In the next phase (current study), the data of CFS patients who completed the treatment programme as well as the TEC, the CIS and the SF-36 at three time points [baseline (pre GCBT), treatment completion (post GCBT) and 1-year follow-up (1-year post GCBT)] were selected. Data collection (RR) and formatting of the data files and data analysis (MV) were performed separately to prevent therapeutic interference. For the current study protocol, an additional approval was obtained by the Medical Ethics Committee of the Antwerp University Hospital (file code 18/19/243). Participants were all over 18 years of age and gave their written informed consent. The study was carried out in accordance with the latest version of the Helsinki Declaration.

### Intervention

The standardized and structured intervention using a closed group setting, comprised 12 group sessions, 3 individual sessions, and one group session for partners, with each session lasting 2 h and the full programme being delivered in 9 to 12 months; for more details of the session content, see **Table 1**. The intervention was delivered by trained and experienced cognitive-behavioral therapists affiliated with the Antwerp University Outpatient Referral Centre for CFS (EB, SB, JI, and LVH).

**Table 1:** Overview of the standardised GCBT for CFS sessions as delivered in the study.

Session	Content
Session 1	Getting acquainted, discussing expectations, rationale and course of the treatment, agreeing on treatment/work plan, goal setting
	Psychoeducation about CFS
	Symptom inventory
	Holistic theory
	Start diary keeping
Session 2	Discussion of diary notes
	Psychoeducation about new, desired behaviour
	Analysis of precipitating stressors
	Goal setting
	Discussion about consequences for patient’s environment
	Self-management strategies
	New observation/journal form
Session 3	Evaluation of the journal notes
	Discussion of day structure and sleep-wake rhythm
	Analysis of stressors
	Learning theory: operant conditioning
	Function analyses of unwanted behaviour
	Counter-conditioning, change process (goals, stimulus control, response prevention, helping thoughts, journal)
Individual session 1	Evaluation of patient’s within-group functioning
	Recapitulation of holistic theory and goals
	Evaluation and adjustment of desired behaviour
	Analysis of new perpetuating stressors
	Function analyses of unwanted behaviour
	Passing on vision, rationale and agreements to partner
	Discussing problem areas of partner
	Discussing long-term goals and setting intermediate goals
Partner session	Information on clinical views on CFS, rehabilitation, goals and results
	Discussing problems between patient and partner
	Group conversation about how to support patient and vice versa
Session 4	Evaluation: reinforcing behavioural change
	Activity inventory to determine appropriate basic activity level
	Acceptance and commitment therapy (ACT): establishing problems with acceptation and challenging catastrophizing thoughts and cognitions
	Information about negative automatic thoughts, how to recognise and challenge them
	Brainstorming about alternative rational thoughts
Session 5	Evaluation: reinforcing behavioural change and focusing on primary goals
	Information on emotions and stress response
	Inventory of negative emotions linked with CFS—challenging catastrophizing thoughts and agreeing on rational thoughts
	Challenging negative automatic thoughts
	Counter-conditioning
	Coping with relapse
Session 6	Evaluation: reinforcing behavioural change
	Evaluation: challenging negative automatic thoughts—letting new helping thoughts take over
	Inventory of helping and non-helping strategies in case of an emotional-hostage situation
	Discussing an escape plan
	Information on emotional vulnerability—discussing dysfunctional schemes and how to change these
	Relaxation—imagination exercises
Session 7	Establishing basic activity level
	Establishing problems with defining boundaries
	Extending operant conditioning—function analyses
	Extending reconditioning (four steps)
	Discussing problems of postponement
	Homework: Identification of environmental stressors
Individual session 2	Evaluation and reinforcement behavioural change
	Evaluation basic activity level
	Discussing long-term goals—resumption of functions—coping with anxiety and worrying
	Discussing progress in functional capacity
	Discussing activity structure as a function of intermediate goals
	Personal stressors
	Problems partner
	Partner as a coach
Session 8	Evaluation of the self: comparison of baseline and current functional capacity—first signs of recovery
	Environment as stressor/vicious cycle
	Discuss actual examples of environmental stressors
	Transactional analyses (elderly, adult, childlike)
Session 9	Evaluation and readjustment of basic activity level
	Explain phases in rehabilitation
	Information about building up (physical, mental, social) activity levels
	Evaluation of response by patient’s environment
	Information on the importance of enjoyment
	Illustrating and discussing new relaxation options
Session 10	Evaluating control/maintenance of basis activity level—reinforcing self-management
	Evaluating build-up of activities
	Evaluating conscious efforts in terms of relaxation/recreation/ enjoyment
	Problems/questions concerning resumption of functions
Individual session 3	Discussing facilitating factors
	Evaluation and adjustment of build-up basic activity level/reinforcing self-management
	Evaluating current functional capacity
	Discussing long-term goals—resuming functions
	Discussing required/desired support and assistance after rehabilitation
Session 11	Evaluating control/maintenance of basic activity level, systematic activity building, time for recreation/enjoyment
	Evaluating coping strategies/long-term thinking
	Relapse prevention
	Feedback on functioning (elderly, adult, childlike)
	Homework: evaluation own views of rehabilitation process
Session 12	Discussing self-evaluation
	Joint evaluation of the intervention
	Information about self-care

### Dependent Variables

#### Checklist Individual Strength (CIS)

The 20-item Checklist Individual Strength (CIS) was used to measure subjective fatigue and behavioural aspects related to fatigue in the previous 2 weeks, providing the researcher an impression of fluctuations over time (21). Each item is scored on a 7-point Likert scale. Four dimensions are measured: fatigue severity, concentration, motivation, and physical activity. In our study we examined the CIS total score as a dependent variable. This score is obtained by adding the scores to all questions (range 20–140). A score of 27 or higher is an indication for abnormal fatigue. A score of 35 or higher indicates severe fatigue. The scale has good internal consistency (α ≈.90), validity and reliability. The CIS was able to discriminate between patients with chronic fatigue syndrome, patients with multiple sclerosis, and healthy controls and the convergent validity was also satisfying.

#### Medical Outcomes Short Form 36 Health Status Survey (SF-36)

The Short Form-36 (22) is one of the most widely used generic self-report measures of health-related quality of life and consists of 36 items that are structured into eight subscales: physical functioning, social functioning, role limitations due to physical or emotional problems, mental health, vitality, bodily pain, and general health perception. The questionnaire additionally generates two summary scores: the physical component summary and the mental component summary. The scales are scored from 0–100 [transformed scale = (actual raw score—lowest possible raw score/possible raw score range) x 100], with higher scores indicating better health. In our study, we used the 10-item physical-functioning subscale, which measures mobility and the ability to perform physically demanding tasks (i.e. walking a mile, walking a block, bending or stooping, and washing or dressing oneself). The measure has demonstrated strong internal consistency (α ≈ 0.80), validity and reliability (23).

### Independent Variable

The following description of the independent variable was in part adopted from De Venter et al. (15). Eligible participants completed the original Dutch version of the Traumatic Experiences Questionnaire (TEC) (24), a 30-item self-report questionnaire inquiring about 29 types of potential trauma, including criterion-A events of PTSD (25), as well as other potentially overwhelming events. In our present study, we considered five domains of early traumatic experiences: emotional neglect (e.g. being left alone, insufficient affection), emotional abuse (e.g. being belittled, teased, called names, threatened verbally, or unjustly punished), physical abuse (e.g. being hit, tortured, or wounded), sexual harassment (i.e. being submitted to sexual acts without physical contact), and sexual abuse (i.e. having undergone sexual acts involving physical contact). Each item consists of a short description of the event of concern, with all items being preceded by the question: “Did this happen to you?” The TEC format allows the presence and severity of childhood trauma to be assessed using four variables: 1. presence of the event(s); 2. duration of the event(s) (in terms of less or more than 1 year); 3. relationship to the perpetrator [parent or sibling versus other person(s)]; and 4. subjective response [in terms of feeling slightly versus moderately, severely, or extremely traumatized by the event(s)]. The variables are awarded a score of 1 if they apply and a score of 0 if they do not. They are scored per age period in which the event(s) occurred (0–6, 7-12, and 13-18 years). Summated, the trauma-severity scores range from 0 to 12 for four subscales: emotional neglect, emotional abuse, sexual harassment (acts of a sexual nature not involving physical contact), and sexual abuse (unwanted sexual acts involving physical contact). The sum score of the fifth subscale, bodily threat, ranges from 0-21 (0-12: physical abuse; 13-21: additional scores concerning threat of life, pain, and bizarre punishment). The main variable is the total composite score (continuous), i.e. the sum of the item scores of the five subscales. The psychometric characteristics of the TEC have been examined in a population of Dutch psychiatric outpatients, where its internal consistency and test-retest reliability were found to be good, with its scores strongly correlating with the scores on the Stressful Life Experiences Questionnaire (24). The TEC has also been administered to nonclinical samples, where it proved sufficiently sensitive to capture low levels of childhood maltreatment (26).

### Demographic and Clinical Variables

Included sociodemographics were: age, gender, having children and educational level. The Dutch version of the Symptom Checklist (SCL-90) was used to assess psychological symptoms (27). The scale consists of 90 items with eight subscales: anxiety, agoraphobia, depression, somatisation, inadequacy, interpersonal sensitivity, hostility, and sleep difficulties. Level of distress is to be rated on a 5-point scale ranging from “not at all” to “extremely”. The scale’s total score (sum of all subscale scores) provides an overall measure of psychoneuroticism. The Dutch SCL-90 has shown good psychometric properties and it is sensitive to the effects of traumatic experiences, with Cronbach’s alphas for the subscales varying from 0.73 to 0.97 and its test-retest reliability ranging between 0.62 to 0.91 (27).

### Statistical Analyses

Statistical analyses were performed using SPSS, version 24 (IBM, New York). Descriptive statistics were calculated. The outcome measures (CIS total and SF-36 physical functioning) were taken before treatment, immediately after 9 to 12 months CBT and 1 year after the end of the treatment. Independent samples t-test and correlation test (Spearman’s rho) were used to measure the associations between the outcome measures at baseline on the one hand and age, sex, and psychiatric status on the other, as well as the association between childhood trauma and age, sex, baseline fatigue, baseline physical functioning and psychiatric status. If significant, the variables will be included in the models described hereafter. To account for the repeated measures, the influence of childhood trauma (baseline TEC scores) on the effectiveness of CBT in CFS, was conducted using a linear mixed model. Fatigue scores and physical functioning scores were considered as time-dependent outcomes. A random intercept per patients is included to account for the dependency between measurements of the same patient. Childhood trauma and the factor time serve as independent fixed variables and interaction between childhood trauma and time is added to both models. The mixed model can handle missing data due to individuals dropping out of the study or selectively completing questionnaires (i.e., can allow for partially complete data) under a missing at random assumption.

## Results

### Study Group Characteristics

The data of 105 consecutive patients who completed the programme as well as the TEC, the CIS and the SF-36 at three time points (baseline (pre GCBT), treatment completion (post GCBT) and 1-year follow-up (1-year post GCBT)) were included and analysed. CIS was completed at the end of treatment and at 1-year follow-up by respectively 94.3% and 87.6% of the patients. Regarding SF-36 the percentages are respectively 96.2% and 87.6%. The baseline characteristics of the study group are presented in **Table 2**. The participants’ smean age was 43.21 years (SD = 7.73), with the greater majority (91.4%) being female and 75.2% having at least one child. Besides CFS, 38.1% of the participants also reported a diagnosis of fibromyalgia and almost half of all participants had suffered a major depressive disorder or an anxiety disorder at least once in their lifetime. Fifty-five per cent had lived through at least one traumatic childhood experience. The scores of our CFS group were comparable to the CFS reference scores reported by Vercoulen et al. (21) (CIS total) (Mean 113.1; SD 14.6) and Crawley et al. (28) (SF-36 physical functioning scale) (Mean 40.6; SD 22.7). Scores on the SCL-90 subscales were compared with the healthy Dutch population (27). Overall our CFS group had worse psychological problems than healthy persons. There was no significant correlation found between childhood trauma on the one hand and baseline fatigue (CIS), baseline physical functioning (SF-36), age, sex, and psychiatric status on the other. The baseline outcome measures were also not correlated with age, sex and psychiatric status. We therefore decided not to include any of these variables in the linear mixed model that will be described in the following section.

**Table 2:** Baseline demographic and clinical characteristics of the study group (N=105).

	N (%) or mean (SD)	
Age in years	43.21 (± 7.73)	
Female	96 (91.4%)	
Having children	79 (75.2%)	
Educational level		
1 Primary school certificate	4 (3.8%)	
2 Lower secondary school certificate	18 (17.1%)	
3 Upper secondary school certificate	46 (43.8%)	
4 Higher vocational/university degree	37 (35.2%)	
Fibromyalgia	40 (38.1%)	
Lifetime major depression or anxiety disorder (SCID-I)	51(48.6%)	
Lifetime major depression (SCID-1)	35 (33.3%)	
Lifetime anxiety disorder (SCID-1)	33 (31.4%)	
Any childhood trauma at baseline	58 (55.2%)	
Emotional neglect	37 (35.2%)	
Emotional abuse	35 (33.3%)	
Bodily threat	17 (16.2%)	
Physical abuse	18 (17.1%)	
Sexual harassment	23 (21.9%)	
Sexual abuse	7 (6.7%)	
Physical functioning [SF-36 (0-100)]	44.76 (± 18.10)	
Fatigue [CIS (7-140)]	114.20 (± 15.68)	
On partial or full disability or unemployed because of illness	74 (70.5%)	
Post-exertion malaise	88 (83.8%)	
Unrefreshing sleep	95 (90.5%)	
Cognitive impairment (subjective)	90 (85.7%)	
Current psychological problems (SCL-90)	CFS study population	Healthy Dutch population
Psychoneuroticism	204.43 (± 43.62)	124.74 (± 33.50)
Anxiety	22.29 (± 6.67)	14.03 (± 5.25)
Agoraphobia	10.79 (± 4.65)	8.42 (± 3.00)
Depression	38.43 (± 11.39)	22.70 (± 7.86)
Somatization	36.08 (± 8.10)	17.95 (± 6.64)
Interpersonal sensitivity	33.65 (± 11.27)	25.70 (± 8.15)
Hostility	9.8 (± 3.51)	7.56 (± 2.44)
Inadequacy	28.10 (± 6.54)	13.78 (± 4.93)
Sleep difficulties	9.50 (± 3.23)	5.00 (± 2.67)

SCID-1, Structured Clinical Interview for DSM-IV Axis Disorders; SCL-90, Symptom Checklist.

### Effect of Childhood Trauma on Treatment Outcomes

**Table 3** summarises the results of the statistical analyses, with each dependent variable shown separately. There was no significant effect of childhood trauma on fatigue or physical functioning in CFS patients, implying that the response to GCBT was similar in CFS patients regardless the childhood trauma score. There was a significant main effect of time on the response of GCBT in CFS patients, indicating a reduction of fatigue scores and an improvement in physical functioning across time. Finally, there was no significant interaction effect between childhood trauma and time, so childhood trauma has no effect on the evolution of fatigue or physical functioning scores after GCBT in CFS patients.

**Table 3:** Summary of the results of the linear mixed model analyses.

	Unstandardized coefficient	Standard error	p-value
*CIS total*			
Time (reference category = pre GCBT)			<0.001
Time = post GCBT	-9.16	2.54	<0.001
Time = 1-year post GCBT	-11.26	2.62	<0.001
TEC	0.28	0.17	0.10
TEC X Time (reference category = pre GCBT)			0.42
TEC X Time = post GCBT	-0.22	0.17	0.20
TEC X Time = 1-year post GCBT	-0.14	0.18	0.43
			
*SF-36 physical functioning*			
Time (reference category = pre GCBT)			0.026
Time = post GCBT	3.34	2.18	0.13
Time = 1-year post GCBT	6.06	2.24	0.007
TEC	0.17	0.17	0.31
TEC X Time (reference category = pre GCBT)			0.27
TEC X Time = post GCBT	-0.05	0.15	0.73
TEC X Time = 1-year post GCBT	-0.24	0.15	0.12

CIS, Checklist Individual Strength; SF-36, Medical Outcomes Short Form 36 Health Status Survey; TEC, Traumatic Experiences Checklist.

## Discussion

### Main Findings

We examined the impact of childhood trauma on the response in fatigue and physical functioning after group CBT in adults coping with chronic fatigue syndrome. We found no effect of childhood trauma on these outcome measures. In other words, the level of trauma experienced in childhood does not influence the effect of GCBT on fatigue and physical functioning in CFS patients. The factor of time, on the other hand, seems to be significantly associated with response of GCBT. Overall, levels of fatigue decreased while physical functioning scores increased across time. Finally, the lack of a significant interaction effect between childhood trauma and time indicated the absence of an effect of childhood trauma on the evolution of fatigue or physical functioning scores after GCBT in CFS patients.

### Comparison With Existing Literature

The results of our study correspond to those of Heins et al. who also did not find any differences in self-reported fatigue levels following individual CBT and again after 6 months between CFS patients with or without a history of childhood maltreatment (18). In contrast to their dichotomous approach and strict cut-off scores for childhood maltreatment, we employed a more continuous approach to childhood trauma. Moreover, the questionnaire we used to qualify and quantify childhood trauma also gauged the emotional experience of the reported adverse event(s) (24). The two studies furthermore show that neither in an individual nor in a group format treatment responses were affected by childhood trauma. This was also the conclusion of Janse et al. (6), who compared the long-term (± 10 years) outcomes of individual and GCBT with a stepped-care programme (a minimal intervention based on the original CBT protocol). Wiborg et al. (5) had earlier concluded that group size does not seem to affect the general efficacy of CBT either.

Overall, there was a positive evolution of fatigue and physical functioning immediately after GCBT and at 1-year follow-up. In their recent follow-up study, Janse et al. also observed that positive post-CBT change scores on these two measures had been partly maintained after 18 months (6).

Looking at the effect of childhood trauma on the efficacy of CBT in other disorders, we noted similar results. Bruce et al. reported that childhood maltreatment was not related to the response rate to CBT in individuals with social anxiety disorder, but that victims of emotional and sexual trauma did report more severe pre- and post-treatment symptoms and/or deterioration (29). Investigating the impact of childhood abuse and childhood neglect (CTQ-SF) on the outcome of GCBT for panic disorder, Santacana et al. likewise found no effects for these trauma-related variables (30). On the other hand, according to the TADS (Treatment for Adolescents with Depression Study), depressed teens with a traumatic past responded worse to CBT than those without (31). Then again, in a study evaluating CBT for deliberate self-harm, patients with a history of childhood sexual abuse showed a more favourable treatment response. It is suggested that this subgroup may have gained more from the treatment because of the overlap in the childhood-trauma and self-harm experiences. It needs to be noted, though, that the patients were offered a combination of CBT and treatment as usual, leaving it unclear to which extent the positive results could be attributed to specific ingredients of the CBT (32). According to Craighead and Nemeroff (33) treatment effects in patients with a history of childhood sexual abuse or parental loss are stronger when a treatment concurrently addresses several dimensions of the problem. In a meta-analysis of psychological treatments for posttraumatic stress disorder in adult survivors of childhood abuse, results showed that trauma-focused treatments were more efficacious than non-trauma-focused interventions. They concluded that the best effects can be achieved with individual trauma-focused treatments (34).

### Limitations

Several limitations of our study warrant attention. In this study the diagnosis of CFS has been confirmed according to the 1994 case definition criteria by Fukuda et al. (1). These criteria have been criticized, because post-exertional malaise and cognitive symptoms are not required for the diagnosis, although these are considered to be core symptoms (3). In a recent study, post-exertional malaise was associated with a greater symptom burden and psychological distress in patients diagnosed with CFS (35). However, the new diagnostic criteria of Myalgic Encephalomyelitis/CFS (SEID) were published in 2015 by the Institute of Medicine (National Academy of Medicine) (3, 4) and all our data have been collected before 2015. Therefore, we could not adopt these new criteria in the current study. In our sample, 83.8% (n=88) of the patients reported post-exertional malaise, 90.5% (n=95) unrefreshing sleep and 85.7% (n=90) cognitive impairment, but we did not take orthostatic intolerance into account.

Furthermore, our indices of treatment outcome were restricted to self-reported and thus subjective levels of fatigue and physical functioning. Although these are core features in the diagnosis and treatment of CFS and the two main outcome measures in most follow-up studies, other characteristic symptoms such as post-exertional malaise, duration and quality of sleep, muscle pain or memory deficits may also be informative and taken into account. As we did not collect data on the course of these latter symptoms and other fatigue influencing parameters, we cannot rule out that these and other treatment-related outcome variables may have been affected by childhood trauma.

Moreover, because of the limited sample, we could not examine the interaction of childhood trauma subtypes, nor the interaction of CFS subtypes on the treatment outcomes. In the follow-up study by Collin et al. (36) for instance, multiple symptoms or pain symptoms were associated with less favorable treatment outcomes in adult CFS patients (in comparison with CFS patients with few symptoms).

We also did not specifically consider life stresses or trauma having occurred in adulthood either. Such experiences may have mediated the relationship between the reported childhood adversity and the post-treatment and follow-up changes in our CFS sample. The Traumatic Experiences Questionnaire (TEC) does not include data concerning persisting symptoms or behavioral changes due to the trauma experienced in childhood. So in this study we did not take the impact of childhood trauma on present life into account. We did assess current psychological symptoms with SCL-90 at baseline, but we did not *a priori* consider SCL-90 (total score or subscale scores) as primary outcome measure nor did we systematically collect these scores as follow-up data.

CFS is more prevalent in women, but the prevalence in the current study (N=96; 91.4%) was higher than expected (37). It is possible that women are more willing to participate in GCBT programmes than men tend to be.

And finally, we inevitably exclusively relied on retrospective self-reports of childhood trauma. Yet, although a review of studies using retrospective accounts of major adverse childhood experiences indeed showed a memory bias, this bias was never sufficient to invalidate the reports (38).

### General Conclusion and Recommendations for Future Research

Childhood trauma does not affect the response to group CBT for CFS in terms of improvement of fatigue and physical functioning. The results of our and earlier studies imply that in the selection of participants for individual or group CBT for CFS, having a history of childhood trauma should not be an exclusion criterion.

However, although childhood trauma is not related to the treatment response of group or individual CBT for CFS, Heins et al. (18) found that patients with a history of childhood maltreatment did report more psychological symptoms both before and after CBT. Therefore, problems related to the childhood trauma could be more persisting even after cognitive behavioral therapy developed to treat CFS symptoms. It is possible that CFS patients with a history of childhood trauma should first obtain a certain amount of physical and mental resilience (through CBT), before engaging in a physically and mentally demanding trauma focused therapy. Examining the effect of the combination with more specific trauma focused therapies might be an interesting recommendation for future research in these patients.

Further research is required to determine the exact influence of gender on the predictive value of childhood trauma on pre- and post-therapy levels of fatigue and physical functioning in the CFS population, as well as the differential effects of CFS subtypes and of childhood-trauma subtypes. Additionally, persisting symptoms, behavioral or personality changes later in life due to childhood trauma could also moderate this relationship. Finally, given the growing reservations about the efficacy of (individual or group) CBT for CFS, it also seems relevant to look whether and to what extent childhood trauma moderates the response to other types of psychological treatments such as psychodynamic therapy, experiential therapy and trauma-focused interventions.

Finally, in the light of the ongoing debate about the remarkable differences between the treatment effects reported for groups and individual patients, besides focusing on general factors that are likely to adversely affect treatment outcome, it also is highly relevant to assess the role of patient-specific predisposing and protective factors for CFS to enable us to tailor interventions to subgroups and individual patients.

## Data Availability Statement

All datasets generated for this study are included in the article.

## Ethics Statement

The study protocol was approved by the local ethics committee of the Antwerp University Hospital (file code 18/19/243). Participants were all over 18 years of age and gave their written informed consent. The study was carried out in accordance with the latest version of the Helsinki Declaration.

## Author Contributions

All authors made substantial contributions to the study. MV and FV developed the objectives of the study. JI, RR, GM, and FV performed the acquisition of data. MV performed the analysis. MV and FV provided interpretation of the data. All authors contributed to the drafting and reviewing of the manuscript. All authors provide approval for publication of the content and agree to be accountable for all aspects of the work in ensuring that questions related to the accuracy or integrity of any part of the work are appropriately investigated and resolved.

## Conflict of Interest

JI received financial support (non-related to the work reported here) from an external funding organisation (registration number EF 84900H bequest M. Lauwers) through GM.

The remaining authors declare that the research was conducted in the absence of any commercial or financial relationships that could be construed as a potential conflict of interest.
